# Fast extraction of three-dimensional nanofiber orientation from WAXD patterns using machine learning

**DOI:** 10.1107/S205225252300204X

**Published:** 2023-03-25

**Authors:** Minghui Sun, Zheng Dong, Liyuan Wu, Haodong Yao, Wenchao Niu, Deting Xu, Ping Chen, Himadri S. Gupta, Yi Zhang, Yuhui Dong, Chunying Chen, Lina Zhao

**Affiliations:** a Multidisciplinary Initiative Center, Institute of High Energy Physics, Chinese Academy of Sciences, Beijing 100049, People’s Republic of China; b University of Chinese Academy of Sciences, Beijing 100049, People’s Republic of China; c School of Engineering and Material Science, Queen Mary University of London, London E1 4NS, United Kingdom; d National Center for Nanoscience and Technology of China, Beijing 10084, People’s Republic of China; UCL, United Kingdom

**Keywords:** machine learning, synchrotron microfocus X-ray diffraction, biological materials, nanofiber networks

## Abstract

Structural disclosure of biological materials can help our understanding of design disciplines in nature and inspire research for artificial materials. Synchrotron microfocus X-ray diffraction is one of the main techniques for characterizing hierarchically structured biological materials, especially the 3D orientation distribution of their interpenetrating nanofiber networks. However, extraction of 3D fiber orientation from X-ray patterns is still carried out by iterative parametric fitting, with disadvantages of time consumption and demand for expertise and initial parameter estimates. When faced with high-throughput experiments, existing analysis methods cannot meet the real time analysis challenges. In this work, using the assumption that the X-ray illuminated volume is dominated by two groups of nanofibers in a gradient biological composite, a machine-learning based method is proposed for fast and automatic fiber orientation metrics prediction from synchrotron X-ray micro-focused diffraction data. The simulated data were corrupted in the training procedure to guarantee the prediction ability of the trained machine-learning algorithm in real-world experimental data predictions. Label transformation was used to resolve the jump discontinuity problem when predicting angle parameters. The proposed method shows promise for application in the automatic data-processing pipeline for fast analysis of the vast data generated from multiscale diffraction-based tomography characterization of textured biomaterials.

## Abstract 2

A machine-learning (ML) based framework can help to unveil the nanofiber orientation in hierarchically structured biological materials with the advantages of fast speed and automation. It promises to meet the analysis demand of extensive amounts of related data online and in real time in near-future next-generation synchrotrons.

## 1. Introduction

Many biological, bioinspired and synthetic materials exhibit 3D networks of textured nanofibers, especially for high-strength and multifunctional materials containing nanofibrillar constituents (Ma *et al.*, 2020 ▸; Zhang *et al.*, 2014 ▸, 2021 ▸; Kargarzadeh *et al.*, 2017 ▸; Peng *et al.*, 2020 ▸; Meyers *et al.*, 2008 ▸). Their key functionality and properties are closely related with nanofiber orientation (Mittal *et al.*, 2018 ▸; Meyers *et al.*, 2008 ▸). The accurate and fast characterization of nanofiber orientation will help to reveal important structural information, elucidate the relationship between structure and property, and thereby provide a way for material modification (Li *et al.*, 2015 ▸) and bioinspired material design (Amorim *et al.*, 2021 ▸).

Synchrotron small-angle X-ray scattering (SAXS) and wide-angle X-ray diffraction (WAXD) methods are widely employed to rapidly and non-destructively extract the orientation distribution information of nanofiber-based composites. Though the texture information of 2D-layered nanocomposites can be directly acquired by fitting the SAXS/WAXD peaks, the determination of the orientation information in the natural nanocomposite is usually not straightforward due to their hierarchical structures. Usually, reconstruction of the complex fiber orientation within composite material requires well designed mathematical models to extract information from different measurement sensors (Breuer *et al.*, 2019 ▸; Chung & Kwon, 2002 ▸; Jack & Smith, 2005 ▸). To tackle this, a mathematical model of the ultrastructure based on the diffraction geometry has recently been developed to reconstruct the 3D information in reciprocal space from 2D experimental SAXS/WAXD patterns, and hence retrieve 3D orientation parameters of the nanofibers inside the X-ray illuminated volume (Zhang *et al.*, 2016 ▸). Although this analytical approach has demonstrated success in retrieving nanofiber orientation information from SAXS/WAXD data collected from hierarchically structured biological tissues like mantis shrimp cuticle, wood *etc.* (Rennhofer *et al.*, 2019 ▸; Rosén *et al.*, 2018 ▸; Lichtenegger *et al.*, 1999 ▸), as we know, there are some inevitable shortcomings. First, the present fitting algorithm is still very time-consuming (*ca* 3 min for each sample), which makes online analysis impossible. Second, it requires domain knowledge of the sample structure and X-ray diffraction, which hampers non-experts. Third, the results are highly dependent on initial parameter estimates that differ between different analyzers, thereby leading to variation. With the advances in beamline instrumentation (Liebi *et al.*, 2018 ▸, 2021 ▸, 2015 ▸), the combination of SAXS/WAXD characterization with tomography techniques has gained great popularity to perform a multiscale study on the sample. For heterogeneous biological tissues, the SAXS/WAXD tomography experiments require a 4D or 5D scan across real and reciprocal space to fulfill the requirements of rotational invariance, resulting in an explosion of experimental data. In fact, a full dataset for the cutting-edge tensor X-ray tomography or 6D SAXS tomography may contain millions of scattering or diffraction patterns in the new-generation beamlines. Therefore, it is foreseeable that the real time data analysis of future SAXS/WAXD tomography experiments will face great challenges which cannot be resolved using the existing method. The development of a novel method for fast and automatic analysis of these enormous datasets is essential.

Machine-leaning (ML) based approaches have generally been believed to be a necessary means for fully exploiting the powerful experimental strength of next-generation synchrotrons (Li *et al.*, 2021 ▸; Dong *et al.*, 2022 ▸). Fitting large SAXS/WAXD tomography datasets with millions of patterns will require the development of a highly parallel program. For traditional modeling, although a parallel fitting program can be developed, the optimized physical model requires human experience to choose the input parameters, which severely limits the batch-fitting ability. In comparison, with ready implementation in parallel computing, ML will have the unrivaled advantage in a batch-fitting task of hundreds of thousands of diffraction patterns because no human experience is required. Until now, the ML-based method has not yet been reported for synchrotron diffraction data analysis of nanofiber systems, though there have been a number of helpful studies on ML applications in related fields, mainly X-ray adsorption spectroscopy (XAS) and X-ray diffraction (XRD) (Wang *et al.*, 2020 ▸; Timoshenko *et al.*, 2019 ▸, 2018 ▸; Carbone *et al.*, 2020 ▸; Oviedo *et al.*, 2019 ▸; Dong *et al.*, 2021 ▸). These works have different characteristics in terms of neural network (NN) structure and data processing methods. First, a NN structure is essential in ML applications. A customized deep convolutional neural network (CNN) has been developed as a tool for property analysis of multi-phase systems, where the same features extractor (*i.e.* CNN module) was shared for subsequent multi-tasks (Dong *et al.*, 2021 ▸). A message-passing neural network (MPNN), a kind of graph-based NN that is most appropriate for modeling molecules, was used in predicting molecular X-ray absorption near-edge spectroscopy spectra on the basis of the oxygen- or nitro­gen-containing molecular structures from the QM9 dataset and achieved quantitative accuracy (Carbone *et al.*, 2020 ▸). Second, and most significantly, owing to the scarce experimental data, almost all algorithms are trained on simulated datasets, and therefore an appropriate data-transformation strategy is needed to guarantee the consistency between experiment and simulation. Wang *et al.* (2020 ▸) extracted noise signals from experimental data and diffraction signals from simulated data, and used them to synthesize the training data for rapid classification of metal–organic framework (MOF) materials with XRD patterns as input. Oviedo *et al.* (2019 ▸) achieved fast identification of the crystalline dimensions and space group. To adapt to the characteristics of thin-film materials, diffraction peaks in the simulated XRD spectra were scaled, eliminated randomly and shifted as a specific means of data transformation. Third, dimensional reduction can be utilized to remove uncorrelated/low-correlated features and make the prediction robust. Timoshenko *et al.* (2019 ▸, 2018 ▸) employed an NN approach to extract the partial radial distribution function (PRDF) with extended X-ray absorption fine structure, thereby characterizing the b.c.c. to f.c.c. phase transformation temperature of bulk iron and exploring the catalyst mechanism of monometallic and bimetallic metal nanoparticles (NPs). To summarise, a proper network structure, data transformation strategy and dimension reduction could be effective factors in the present diffraction data analysis. The ML-based method requires development for the analysis of synchrotron-based nanofiber diffraction data, but resolving nanofiber diffraction data is a multi-targets regression task and is extremely different from the XRD or XAS data mentioned above, including marginally separated diffraction peaks and circular adjacent features. Therefore, a specific ML scheme is demanded for the successful nanofiber orientation prediction.

**Figure 1:**
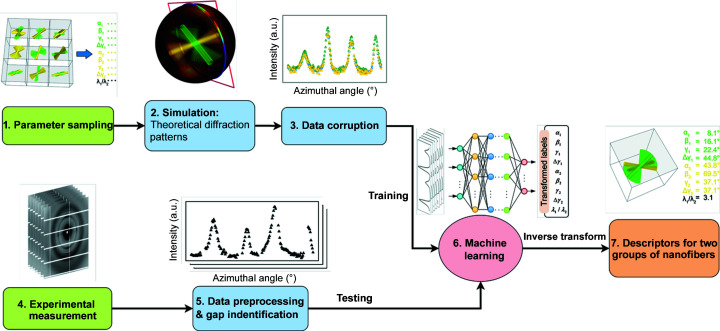
Schematic of the fiber orientation prediction framework for two groups of nanofiber systems with data corruption on the simulated dataset.

In this work, under the assumption that two sets of well separated nanofibers are distributed across the examined sample with changing local orientation parameters, we demonstrate an NN method for the orientation metrics identification of 3D textured nanofibers based on synchrotron X-ray diffraction *I*(χ) data. Data corruption and orientation label transformation are implemented for resistance to experimental noises, ensuring predictive performance for experimental data with various missing regions and resolving the jump discontinuity problem when predicting angle labels. The framework used for automatic and fast orientation prediction of 3D textured nanofibers is shown in Fig. 1 ▸. There are six types of ML algorithms utilized here for pursuing the best fit. These ML algorithms are trained and validated on the simulated data generated from the physical nanofiber diffraction-scattering model from the previous results of our group (Zhang *et al.*, 2016 ▸; Zhang, De Falco *et al.*, 2017 ▸). The hierarchically structured stomotopod (mantis shrimp) cuticle is endowed with great impact resistance by its interpenetrating chitin nanofibers and is therefore a popular biological material model for bionic research. To test its effectiveness in real-world data, the method is applied to analyze experimental diffraction data collected from a stomatopod telson cuticle. Since most synchrotron facilities are building artificial intelligence (AI) platforms to overcome the barriers associated with using cutting-edge ML algorithms and computing resources for future users, developing a customized ML analysis pipeline will become increasingly easy (Dong *et al.*, 2022 ▸). The development of the proposed method therefore represents a big step forward to a highly automatic data-processing pipeline for future high-throughput and multidimensional WAXD characterizations.

## 2. Experimental

### 2.1. Theoretical data generation and ML algorithms used

**Figure 2:**
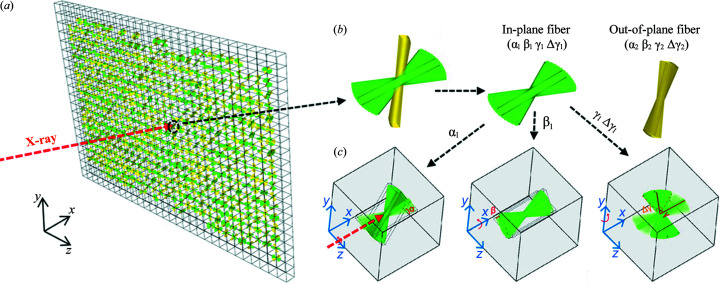
Schematics showing (*a*) a sectioned specimen illuminated by a micro-focused X-ray beam, (*b*) in-plane (green) and out-of-plane (gold yellow) fibers, and (*c*) the geometric definition of parameters α, β, γ, Δγ for each group of nanofibers. Note there is also a parameter λ which indicates the quantity for every group of nanofiber, and thereby the scale ratio parameter is derived using λ_1_/λ_2_.

Like other nanofiber-based biological tissues, the hierarchically arranged mineralized chitin fiber networks play a crucial role for the stomatopod cuticle to achieve both high stiffness and high toughness (Yaraghi *et al.*, 2019 ▸; Zhang *et al.*, 2020 ▸). Most regions across the depth of the stomatopod cuticle contain two groups of fibers. A schematic is given in Fig. 2 ▸. One in-plane fiber group is parallel to the morphological surface, forming a twisted plywood structure (known as a Bouligand structure), and one out-of-plane fiber group is oriented perpendicular to the surface [Fig. S2(*b*)] inside the pore canal systems (Zhang *et al.*, 2016 ▸). Generally, two separate pairs of diffraction arcs can be observed in the (110) diffraction region of WAXD patterns collected from a stomatopod cuticle [Fig. S2(*c*)]. In our previous paper, we developed a mathematical model to retrieve 3D information of the fiber groups within the sample from a single (110) diffraction pattern from of the 2D WAXD image. The model is based on diffraction geometry and the fiber symmetry feature in the assembly of nanofibrils into nanofibers. Using the model, we managed to reconstruct the 3D reciprocal *QS*(110) sphere and retrieve two sets of fiber orientation parameters by exploring the complexity in the radially integrated *I*(χ) curve of the (110) diffraction pattern (Zhang *et al.*, 2016 ▸). As described in our published paper, we employed a nonlinear iterative fitting method to fit the experimental *I*(χ) curve to obtain the fiber orientation parameters.

For developing a faster and more accurate fiber orientation prediction method using ML, we simulated vast amounts of synthetic data to train the ML model. In practice, 100 000 orientation labels were sampled, covering all possible orientations of two groups of nanofibers (Table S1 and Figs. S6 and S7 of the supporting information). The developed mathematical model for nanofibers was then used to generate the corresponding *I*(χ) curves. The nanofiber diffraction model is described in Section S2 of supporting information.

The task of ML is to directly obtain nine fiber orientation parameters from the corresponding *I*(χ) curves. Three deep-learning algorithms were implemented to learn the nonlinear relationship between the diffraction pattern and the corresponding targets, including fully connected neural networks [FCNN (Zhang, Lee *et al.*, 2017 ▸; Kingma & Ba, 2014 ▸)] and two CNN models [*i.e.* 1D variants of DenseNet (Huang *et al.*, 2017 ▸) and PreActResNet34 (He *et al.*, 2014 ▸) for adaptation to 1D input]. Their details and architecture are described in Section S1 and Fig. S1 of supporting information. In brief, the objective function is the mean absolute error (MAE) between nine orientation labels and NN output values, and the Adam optimizer (Kingma & Ba, 2014 ▸) is used for minimization. What is more, as representatives of classical ML algorithms, K-nearest neighbors (KNN), random forest (RF) and support vector regression (SVR) were also tested. Bayesian optimization is applied to search the optimal hyperparameters via *Scikit-learn* (Pedregosa *et al.*, 2011 ▸). The optimal hyperparameters for the ML algorithms involved are listed in Table S2. The ML algorithms are implemented through *Scikit-learn*/*Pytorch* software (version 1.6; Paszke *et al.*, 2019 ▸) and run on the Intel Core i9-9900X 3.50 GHz CPU and Nvidia RTX 2080 ▸ super graphic processor. For the sake of the randomness in weight initialization and dataset splits, ML algorithms are trained five times and the results are averaged.

### 2.2. Experimental WAXD data for algorithm verification

Generally, the WAXD patterns were produced after a beam of X-rays with sufficient intensity transmitting through the sample as shown in Fig. S2(*a*). In this paper, the WAXD experiment was conducted at beamline I22 with a monochromatic X-ray beam (beam size 10 × 12 µm) of 10 keV with a flux of about 3.5 × 10^12^ photons s^−1^ at Diamond Light Source (Harwell, UK). During the test, the sample-to-detector distance was set to 262.3 mm. The WAXD patterns were acquired with a Pilatus P300k-W detector (silicon hybrid pixel detector, DECTRIS Ltd, Baden–Daettwil, Switzerland) with a pixel size of 172 × 172 µm and a 0.5 s exposure time. The gaps and bad pixels in the patterns were automatically masked by the threshold (less than 0 and higher than 100 000). A circular mask was further manually added to cover the beamstop and direct beam. The specimens were dissected from mantis shrimp (purchased from the Tropical Marine Centre, London) and sectioned with 1 mm thickness under constant irrigation using a low-speed diamond saw (Buehler Isomet, Buehler, Duesseldorf, Germany). The experiment pattern recorded by a Pilatus P300k-W detector is shown in Fig. S2(*c*).

There are 40 experimental WAXD patterns collected from two separate synchrotron experiments at different beam times (21 images from beam time I and 19 images from beam time II) on thin slices (0.8–1 mm thick) of stomatopod telson cuticles. It has been demonstrated that, only by exploring the intensity variation of the radially integrated *I*(χ) profiles (1D) from the single meridional (110) reflection, we can extract the desired 3D fiber orientation information in the X-ray illuminated sample volume. We also performed radial integration on the (110) reflection of the WAXD patterns using *Fit2D* (Hammersley, 1997 ▸) and a further ring-subtraction described in our previous paper for background removal (Zhang *et al.*, 2016 ▸). Standard nonlinear Levenberg–Marquardt fit algorithms were used to fit the experimental scattering profiles [Fig. S2(*d*)] to obtain the orientation information for the traditional fitting method.

### 2.3. Saliency mapping

Saliency maps were first proposed by Simonyan *et al.* (2013 ▸), and this technique has been widely used in the field of deep-learning interpretation (McGovern *et al.*, 2019 ▸). The explanation for this technique is relatively straightforward. As we know, the regression coefficients indicate the degree of importance of their corresponding features for linear regression. In the case of deep NNs used here, the NN output *S*(*I*) is a highly nonlinear function of input features *I*, whereas *S*(*I*) can be approximated with a linear function through its first-order Tayler expansion:

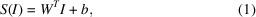

where *W* is the derivative of *S* with respect to the input *I* at point *I*_0_ and *b* is the bias.

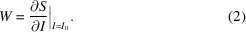

Thus, the absolute values of the elements in *W* will naturally represent the influence rank order of input features on the NN output *S*(*I*).

### 2.4. Evaluation metrics of algorithm performance

In our previous paper, we developed a mathematical model based on the physical diffraction geometry to describe the relationship of 3D nanofiber orientation to its diffraction patterns, and on this basis, 3D nanofiber orientation parameters were retrieved from a single experimental *I*(χ) curve at fixed *Q* [*i.e.* on *QS*(110)] with the help of a nonlinear iterative fitting algorithm. However, even though the traditional model-fitting method based on our mathematical model demonstrated success in retrieving the 3D nanofiber distribution information within the examined cuticle sample, the method still has its limitations: (*a*) the accuracy of the fitting depends on the variable step size when looped over nine parameters, the heavy computational task results in greater time consumption, hence there is a trade-off between prediction accuracy and speed; (*b*) in a real world situation, the nanofiber orientation distribution within the sample can never be perfectly described by a mathematical model, therefore even though the physical model can be considered correct, the experimental *I*(χ) curve can never be fitted with 100% accuracy. Thus, the retrieved nanofiber orientation cannot be considered as the ground truth (*i.e.* unlike the simulated dataset, we have no access to the ground truth labels for experimental data).

For both ML and traditional fitting methods, we evaluated the accuracy by calculating the root mean squared error (RMSE) in equation (3) between the experimental and reconstructed *I*(χ) curves, with the latter generated from the physical model using the nine predicted orientation parameters. The reconstruction process is described schematically in Fig. S9. That is to say, we assessed the algorithm performance in terms of goodness of fit between the reconstructed curve and the original *I*(χ) curve. The peak location/intensity of those 1D *I*(χ) curves could also be evaluated owing to their strong connection to the nanofiber orientation parameters and can be viewed as a real world expression of these hidden parameters. To make a fair comparison, the performance on the simulated dataset is also evaluated in the same way, although the simulated dataset has ground truth labels. Based on these, Table 1 ▸ shows the ML model performance on both the experimental and the simulated datasets in terms of matches of the reconstructed and original *I*(χ) curves, with metrics including the RMSE in equation (3) and the peak-related Pearson coefficient in equation (4). The results are analyzed in the Results and discussion[Sec sec3].

In addition, because the simulated dataset has ground truth orientation parameters, we also evaluate the FCNN performance in terms of the nine ground truth labels (*i.e.* in Fig. 4). Specifically, two additional metrics are calculated, including the coefficient of determinant *R* ^2^ defined in equation (5) and the mean absolute error (MAE) defined in equation (6) between the predicted values and ground truth labels:

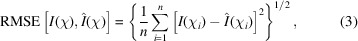




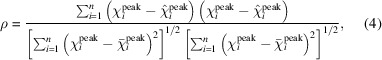




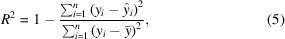




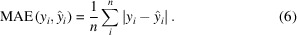




## 3. Results and discussion

### 3.1. Framework for nanofiber orientation prediction

As shown in Fig. 1 ▸, there are six ML algorithms used for learning the mapping from input features to transformed target labels. The label transformation is described in detail in Section 3.2. Our method utilizes simulated diffraction patterns to train an ML algorithm for the orientation-prediction task. Then, the trained ML is subsequently applied to experimental data for testing purposes, which can be challenging because there are no experimental data in the training dataset.

In our previous work, on the basis of the nanofiber diffraction model, a nonlinear iterative fitting method was used to retrieve the 3D nanofiber orientation information from experimental *I*(χ) profiles of the acquired WAXD patterns (Zhang *et al.*, 2016 ▸). However, there are some limitations that block the achievement for high accuracy, high-throughput, online and real time analysis, including required expertise and an initial guess, and the inevitable tradeoff between time efficiency and accuracy. In this work, nanofiber orientations within biological cuticles are allowed to be rapidly and automatically obtained without the participation of domain experts and with a compromise between time consumption and tolerant errors.

In response to measured experimental noises and random missing regions in experimental diffraction patterns, a data corruption strategy is applied to the simulated dataset (details in Section 3.2) with the aim of generalized and robust predictive ability for experimentally measured data. The experimental data were also preprocessed (details in Section 2.2). In brief, this work uses a simulated dataset for training and validation, and experimental datasets are used for testing, where the performance proves that this method is indeed feasible for practical data.

Although only two distinct orientations are considered here, the framework can be extended to tackle multi-group cases by training many models for each of them, like in the published work on XRD phase-fraction prediction (Lee *et al.*, 2021 ▸). For specific data, the reconstructed RMSEs from these models are compared, then the one with lowest RMSE is selected.

### 3.2. Data corruption and orientation label transformation strategy

**Figure 3:**
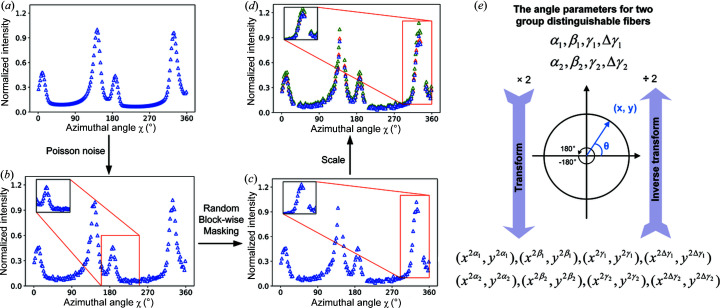
Schematic of data corruption and orientation label transformation. (*a*) Original simulated *I*(χ)_(110)_ curve without noise, (*b*) Poisson noise applied to the simulated *I*(χ)_(110)_ curve, (*c*) random masking of the *I*(χ) curve through a blockwise masking algorithm, (*d*) scaling into a range, (*e*) label transformation which transforms the angle labels into corresponding *x* and *y* coordinates at a fixed radius.

As shown in Fig. 3 ▸, a series of data corruption steps has been implemented to account for the differences between simulated and experimental data, and ensure the prediction ability of the algorithm for experimental data with the natural signal missing and measured noises. At the same time, among labels (*i.e.* orientation parameters), the angle-related labels are transformed into *x* and *y* coordinates with the aim of avoiding jump discontinuity in values during prediction. The data corruption steps for the simulated dataset are shown in Figs. 3 ▸(*a*)–3 ▸(*d*). The label transformation for angle-related labels is shown in Fig. 3 ▸(*e*).

The first issue that needs to be considered is the noise in the experimental data. The peaks in the experimental *I*(χ) profiles are not necessarily standard Lorentz or Gaussian shape. Therefore, the noise cannot be removed by fitting to a standard curve. To ensure the robustness of algorithms to experimentally measured noise, random levels of Poisson noise are added in the simulated data [Fig. 3 ▸(*b*)]. The other two noise settings are also tested, including random Gaussian noise, and random Poisson noise and random Gaussian noise. Table S3 shows the reconstruction results for experimental data and there is no significant difference among them. In addition, the reconstruction results of the algorithm trained on the Poisson noise applied simulation dataset are checked. Fig. S4 shows that the model trained based on the Poisson noise applied simulated dataset is robust to Gaussian noise.

The major and most important difference between the experimental and simulated *I*(χ) profiles is the existence of missing regions in the experimental profiles. These gap regions come from the mask setting on the detector gap and beam stop region. However, though the gap regions on the detector are fixed for a specific module, the missing region in the radially integrated *I*(χ) profiles will change due to different experimental setups. The variation of the beam center position, incident X-ray energy and sample-to-detector distance will result in either the shift of the (110) diffraction peaks [Figs. S2(*a*) and S2(*c*)] or the change of the radius of the reflection ring. This means the positions and sizes of the missing gaps in the experimental profiles will be a constant changing factor among different beam times. The work from masked image modelling in the computer vision community shows that the NN model can learn nontrivial and meaningful latent vison representation through masking random patches of the input image (He *et al.*, 2021 ▸; Xie *et al.*, 2021 ▸). Inspired by this, the masked curve training strategy is used in this work to learn the robust representation and guarantee the predictive ability of the model for different experimental data with variable missing regions. Note that the mask tokens are not necessary in our work because the masked regions still exist in the experimental data. As shown in Fig. 3 ▸(*c*), blockwise masking is employed in the *I*(χ) profiles in the simulated dataset. Unlike blockwise masking for images (Bao *et al.*, 2021 ▸), the head and tail of the *I*(χ) curve are in fact connected with each other. Considering this connectivity, the feasible blockwise masking method for *I*(χ) is summarized in algorithm 1 (see below). A block of the *I*(χ) curve is masked each time, and, as a result, produces similar missing patterns to the experimentally measured *I*(χ) curves. To identify an appropriate masking ratio, Fig. S3 shows the FCNN reconstruction error for experimental *I*(χ) curves versus different masking ratios. From these results, we can conclude that, without the masked curves training strategy, the performance on experimental data is poor, and the lowest reconstruction error of 0.09 can be achieved at masking ratio of 0.3. Finally, to compensate signal fluctuation, the maximal intensity is randomly scaled to [1 − 5%, 1 + 5%] instead of general normalization [Fig. 3 ▸(*d*)].[Chem scheme1] 
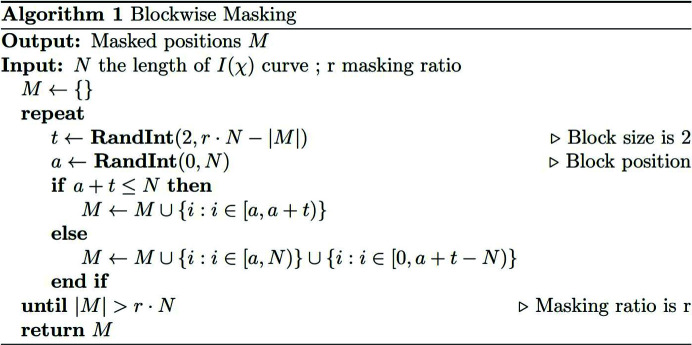


As for orientation labels, two important issues need to be considered. The first is the computational indistinguishability of the two parameter groups which can lead to an unlearnable mapping for algorithms. Specifically, the order of orientation labels for two fiber groups can be switched with each other and not change the corresponding *I*(χ) curves. This means the mapping of algorithms will not be unique. To define a unique mapping for ML algorithms, the order of two fiber groups must be ranked according to the specific rule (*e.g.* descending order of γ magnitude). Herein, we define the fiber group with the larger γ as the first and the other as the second, thus the two fiber groups are artificially distinguishable. Table S4 shows that, compared with the artificially defined distinguishability, the indistinguishability between two groups of fibers leads to an unlearnable mapping.

The other noteworthy issue is that the α_1_, β_1_, γ_1_, Δγ_1,_ α_2_, β_2_, γ_2_ and Δγ_2_ labels have periodicity, but the periodicity is not reflected by their values. For example, physically, β = −90 and β = 90° means the same, yet the values exhibit the largest difference when ranging from −90 to 90°. This means the prediction of algorithms will have jump discontinuity on those labels. As shown in Fig. 3 ▸(*e*), the angle labels are first multiplied by 2 and the corresponding range will be from −180 to 180°, then they are transformed into corresponding *x* and *y* coordinates at a fixed radius (*e.g.* unit circle). This transformation ensures predictive continuity at boundary locations. Although they refer to the same thing, the algorithms will not directly predict angles but transform angles (*i.e.* *x* and *y* coordinates), and the prediction values will be inversely transformed into angles in our implementation.

### 3.3. Machine-learning prediction performance

On account of the difference between the simulated and experimental data, even after data corruption, the ML algorithm performance is evaluated under two phases. Phase I: simulated data are used for both training and testing purposes; phase II: simulated data are used for model training whereas the real world experimental data are used for testing. As stated in Section 2.4, owing to the lack of ground truth orientation parameters for the experimental data, the accuracy of the ML model is evaluated by the goodness of fit between the experimental and reconstructed *I*(χ) curves, with the latter calculated from the physical model using the nine predicted orientation parameters. Table 1 ▸ summarizes the performance metrics of various algorithms under two phases together with the corresponding standard deviations in parentheses. Phase I clearly has a lower RMSE than phase II because training and testing are both based on the simulated dataset, thus ensuring adequate consistency. Compared with phase I, the higher RMSE reveals the discrepancy between the experimental data and the simulated data. The Pearson correlation coefficient for peak locations and peak intensity follows the same trend, but some differences exist. The Pearson correlation coefficient is higher for peak location than that of peak intensity for both phases. Meanwhile, the decrease in the Pearson correlation coefficient for peak intensity is larger in comparison with the peak location from phase I to phase II. This phenomenon implicitly reveals that peak location is more easily learned than peak intensity by ML algorithms.

For both phases, the FCNN demonstrates the best performance compared with the other ML algorithms used. The CNN architectures are generally thought to outperform the relatively simple FCNN in many tasks (Prince *et al.*, 2019 ▸; Oviedo *et al.*, 2019 ▸; Rankine *et al.*, 2020 ▸) because of the parameter-sharing mechanism which reduces the overfitting risk, and utilizes the spatial information efficiently. However the FCNN outperforms the CNN architectures in this study. This discrepancy is mainly attributed to their different assumptions on input data. CNNs makes an assumption that input data has a grid-like structure (Peng *et al.*, 2017 ▸). But the topology and connectivity of original 2D diffraction patterns are not intactly preserved in *I*(χ) curves, thus the assumption of CNNs breaks apart. In comparison, the FCNN is not involved in the inductive bias of CNNs and will learn the actual data topology relationship eventually through training data. In practice, the reconstruction result shows that FCNN can easily pick up the information of different cut-off locations of *I*(χ) curves (Fig. S5).

**Figure 4:**
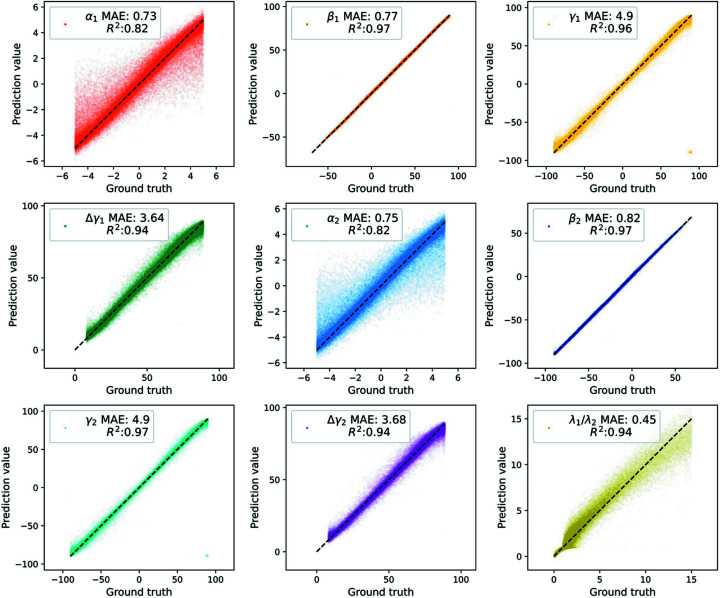
Parity plot of the ground truth and the predicted value with R^2^ and MAE metrics for nine orientation labels (*i.e.* α_1_, β_1_, γ_1_, Δγ_1_, α_2_, β_2_, γ_2_, Δγ_2_ and λ_1_/λ_2_ respectively) on the simulated dataset.

For the performance of FCNN on the simulated dataset, we analyze the parity plot between ground truth and predicted orientation parameters with MAE and *R* ^2^ metrics in Fig. 4 ▸. The *R* ^2^ scores of β_1_ and β_2_ are both 0.97 and their MAE values are 0.77 and 0.82, respectively. The *R* ^2^ scores of α_1_ and γ_1_ are 0.82 and 0.96 and their MAE values are 0.73 and 4.9, respectively. With respect to the λ_1_/λ_2_ label, MAE = 0.45 and *R* ^2^ = 0.94. In brief, among the nine labels, the FCNN model shows the best prediction performance for the β and γ labels with *R* ^2^ up to 0.97, and Δγ_1_, Δγ_1_ and λ_1_/λ_2_ with *R* ^2^ = 0.94, and at last the *R* ^2^ metrics for two α are 0.82. The training loss history of FCNN is shown in Fig. S9.

**Figure 5:**
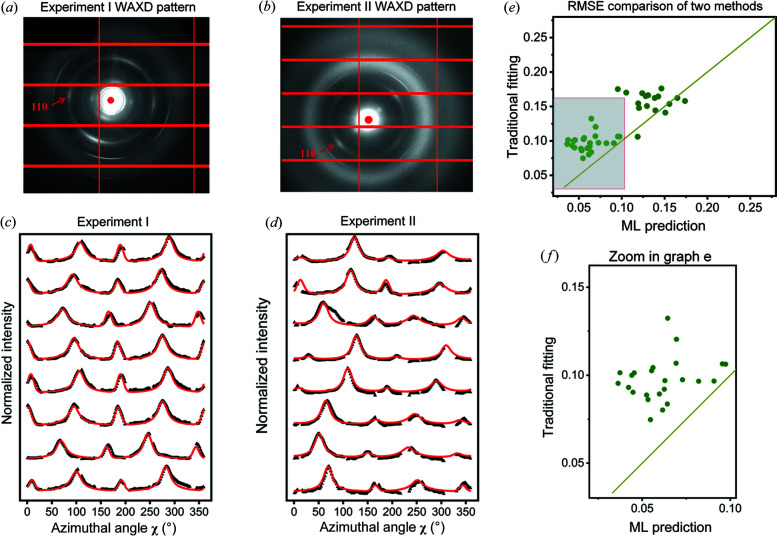
Prediction performance for the FCNN evaluated on two separate experimental datasets from two different experiments. WAXD patterns for experiments (*a*) I and (*b*) II. Waterfall plots of experimental diffraction profiles (black triangle markers) and reconstructed diffraction profiles (red solid line) according to the FCNN prediction values for experiments (*c*) I and (*d*) II. (*e*) Comparison of the reconstruction RMSEs between the traditional iterative fitting and FCNN methods. (*f*) Zoomed-in image of the shaded region of (*e*).

The prediction performances of FCNN on the experimental data [Figs. 5 ▸(*a*) and 5 ▸(*b*)] are shown in Figs. 5 ▸(*c*) and 5 ▸(*d*) by examining the difference between *I*(χ) curves reconstructed from the physical model and experimental curves. Since the training process does not bring in missing signal pattern information of tested experimental data, the reconstructed and experimental curves exhibit a good match for both experiments I and II, which indicates that the trained FCNN model has good compatibility for experimental WAXD patterns collected even from different beamlines with entirely different missing signal patterns. Fig. 5 ▸(*e*) shows the comparison of reconstruction RMSE for the traditional model-fitting method and FCNN for experimental data. The FCNN method achieves a relatively low RMSE value [enlargement shown in Fig. 5(*f*) of the shaded region in Fig. 5 ▸(*e*)]. When the value goes up, the RMSE of the FCNN method will probably surpass the traditional model-fitting method. The result means that when both fitting methods work well, the FCNN performs better. However, when the traditional model-fitting method starts to fail the task, this means fiber orientation distribution in the sample can no longer be well described by the proposed physical model. Under this circumstance, the ML model which is trained with simulation data based on the same physical model cannot be expected to work as well as before.

In conclusion, the general prediction performance of FCNN exceeds the traditional iterative fitting method overall. Since the FCNN fitting method requires no human intervention on setting the fitting parameters of individual data together with excellent parallel capability endowed by parallel matrix computation, it hence has unrivalled advantages in batch-processing and automation capabilities. Besides, in the rare circumstance that large RMSEs (*e.g.* RMSE > 0.17) are obtained, the output parameters from the FCNN model can serve as part of the initial estimates for building the right physical models and accelerate the fitting process in special cases.

**Figure 6:**
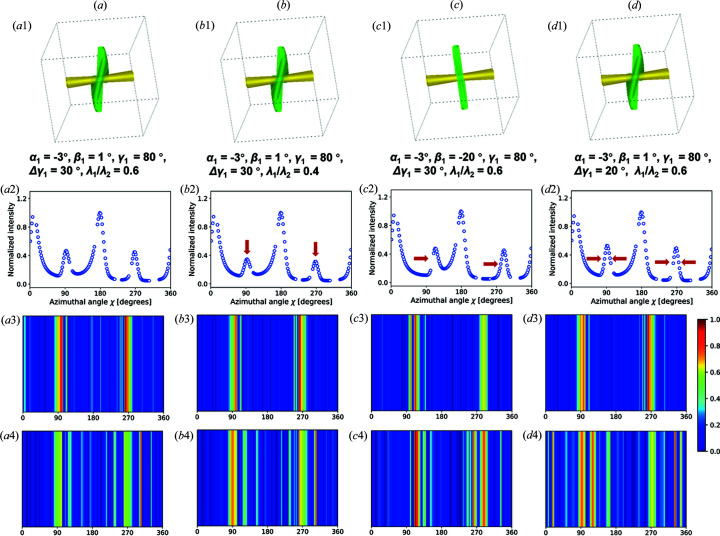
Saliency maps highlighting the features that the FCNN model is dependent on. (*a*)–(*d*) Sketches of two groups of fibers, (*a*1)–(*d*1) one orientation parameter of the specific fiber group (green) changes at each time, (*a*2)–(*d*2) normalized simulated *I*(χ) profile corresponding to the changes in (*a*1)–(*d*1), and the saliency maps (*a*3)–(*d*3) with data corruption and (*a*4)–(*d*4) without data corruption.

Furthermore, we explored the feature dependence of FCNN for nanofiber diffraction analysis. The saliency map is a means to show the saliency of each input feature and the degree of impact that different regions have on prediction. As shown in Fig. 6 ▸, the saliency map for one selected fiber group (green fiber in Figs. 6 ▸(*a*1)–6 ▸(*a*4) highlights highly dependent regions when making predictions with various fiber orientations. With Fig. 6(*a*1) as a comparison, the difference in orientation involves λ_1_/λ_2_ [Fig. 6(*b*1)], β [Fig. 6(*c*1)] and Δγ [Fig. 6(*d*1)], and orientation variance can affect relative peak height, peak shift and full width for half-maximum of diffraction peaks for the variable group of fibers [Figs. 6(*a*2)–6(*d*2)]. Saliency maps for α and γ are shown in Fig. S10. Overall, it suggests that with the present data corruption, the output of FCNN highlights features with a high signal-to noise ratio [Figs. 6(*a*3)–6(*d*3)]. On the contrary, the feature dependency does not show obvious regularity when without data corruption [Figs. 6(*a*4)–6(*d*4)]. The saliency map results suggest that the data corruption strategy focuses the FCNN more on important and robust features and neglect susceptible features (*i.e.* low signal-to-noise).

## 4. Conclusions

Our work first develops an ML framework for the 3D orientation prediction of nanofibers based on the synchrotron diffraction data. Data corruption is applied to the simulated dataset to ensure resistance to experimentally measured noise and predictive ability for experimental data with naturally random missing regions. The transformation for angle-related orientation labels resolves the jump discontinuity problem when predicting angle labels. And this treatment for angle parameters may also be effective for other problems involving predicting angle values. Through saliency analysis, the high-correlation features with prediction are substantially distributed in the peak regions, which is physically meaningful and leads to reliable and robust models. The relationship in the simulated dataset is precisely learned by the FCNN algorithm with a high coefficient of determinant (*R* ^2^ ≥ 0.82). The low reconstruction RMSEs for two separate experiment datasets suggest the practical prediction ability for real-world data with richly varied missing signal patterns. The method can be extended to tackle multi-group nanofiber cases by simple means, *i.e.* training many models for each multi-group case and selecting the one with the lowest error.

Given the merits of automation and rapid speed, this work is a step forward for the future analysis of large amounts of multiscale diffraction characterization data from biological materials. Although more effort is still needed for challenging cases (*e.g.* peak splitting and close orientations), its application will still help to accelerate the past expert-need and time-consuming analysis of fiber orientations. Furthermore, this method possesses great potential to be an online tool so that the real time analysis and experimental adjustments will become possible. Looking to the future, the methods developed in this project are expected to play greater roles for material characterization when integrated into the data acquisition and online data analysis software systems of synchrotron beamlines (Liu *et al.*, 2022 ▸).

## 5. Data and code

Data for this study are available at https://ihepbox.ihep.ac.cn/ihepbox/index.php/s/YH9WWaZCS84dmTN. The demo code in the form of a Jupyter notebook is available at https://github.com/LinaZhaoAIGroup/nanofiber. 

## Supplementary Material

Supporting figures and tables. DOI: 10.1107/S205225252300204X/ro5032sup1.pdf

## Figures and Tables

**Table 1:** Performance metrics of the ML algorithms for two phases Reported RMSEs and peak-related Pearson correlation coefficients in terms of the matches between experimental and reconstructed *I*(χ) curves. Phase I: training and testing data are all simulated. Phase II: real-world experimental data for the cuticle of a stomatopod telson are used for testing. The results are the average of five repetitions together with corresponding the standard deviations in parentheses.

	Phase I		Phase II	
Method	RMSE	Pearson coefficient (peak location/peak intensity)	RMSE	Pearson coefficient (peak location/peak intensity)
K-nearest neighbors	0.1843 (0.0062)	0.9520/0.6717 (0.0111/0.0657)	0.2303 (0.0207)	0.9406/0.6286 (0.0024/0.0937)
Random forest	0.2947 (0.0065)	0.9651/0.372 (0.0026/0.0319)	0.4001 (0.0045)	0.9478/0.3462 (0.0055/0.0220)
Support vector machine	0.2444 (0.0111)	0.9246/0.6764 (0.0041/0.0567)	0.282 (0.0079)	0.9307/0.5250 (0.0062/0.1210)
Fully connected neural networks	0.0265 (0.0024)	0.9982/0.9731 (0.0002/0.0067)	0.0902 (0.0025)	0.9983/0.9349 (0.0012/0.0064)
DenseNet	0.0322 (0.0014)	0.9953/0.9564 (0.0005/0.0027)	0.1332 (0.0072)	0.9915/0.7928 (0.0023/0.0094)
PreActResNet	0.0467 (0.0054)	0.9836/0.9636 (0.0014/0.0043)	0.1991 (0.0173)	0.9795/0.7200 (0.0041/0.0593)
