# Supplementary material for: Fast extraction of three-dimensional nanofiber orientation from WAXD patterns using machine learning
Source: IUCrJ. 2023 Mar 25;10(Pt 3):297–308. doi: 10.1107/S205225252300204X (PMC10161767; doi:10.1107/S205225252300204X)
Supplement: Supplementary file 1 [file m-10-00297-sup1.pdf]

IUCrJ

Volume 10 (2023)

Supporting information for article:

Fast extraction of three-dimensional nanofiber orientation from WAXD patterns using machine learning

Minghui Sun, Zheng Dong, Liyuan Wu, Haodong Yao, Wenchao Niu, Deting Xu, Ping Chen, Himadri S. Gupta, Yi Zhang, Yuhui Dong, Chunying Chen and Lina Zhao

S1. Deep learning architectures

Three widely used DL architectures, i.e., FCNN, PreActResNet and DenseNet are used, because they have different characteristics. FCNN is a basic fully connected neural network. It doesn't rely on any assumption of feature adjacent relationship (i.e., don't depend on specific data structure), but its training needs more data than CNN-based architectures. PreActResNet is a CNN-based residual neural network. It assumes that input features conform to grid structure (e.g., images). The main advantage is that when NN goes deeper its residual connection avoids overfitting, therefore the PreActResNet can learn more complex nonlinear relationship. As a CNN architecture, it consumes less data than FCNN due to efficient utilization on data structure information. DenseNet is also a CNN-based residual neural network, which considers more than first-order residual connection, thus has a stronger fitting ability and less overfitting risks than PreActResNet. However, its significant disadvantage is heavy-computation burden.

The FCNN is composed of 1 input layer, 6 hidden layers in which the first hidden layer contains 2048 neurons and the others have the 30% less neurons than the last layer, as well as an output layer corresponding to 9 output targets. The batch normalization (BN) layer is used between the layers except input layer. After BN layers, ReLU (rectified linear unit function) is used as activation function. The PreActResNet34 comprises of 4 units which include 3, 4, 6 and 3 building blocks respectively, and each block contains 2 convolution layers, total of 34 layers together with input convolution and output transformation layers. The DenseNet implemented is composed of 4 Dense Blocks with 4 convolution layers in each block and plane growth rate equal to 64. To reduce the number of planes, the transition layer is applied between Dense Blocks. The mean absolute error (MAE) is used as objective function and Adam optimizer used to minimize it for all three DNN algorithms. The early stopping technique is applied to identify the best model state before overfitting.

S2. Parametrization of nanofiber orientation and nanofiber diffraction model

Based on the fact that the c-axis of the chitin fibril unit cell exhibit fiber symmetry when assembled into nanofiber, each nanofiber will generate a *(110)* diffraction ring of equal intensity in the reciprocal space (described as a δ-function). The spreading of the nanofiber groups (described as weight function) in the x-ray illuminated sample volume will lead to certain 3D intensity distribution of the QS*(110)* reciprocal sphere, which can be described by a mathematical model combining, the δ-function and weight function. As the in-plane nanofibers within the stomatopod cuticle are arranged in a plywood style interrupted by pore canal with out-of-plane nanofibers, we can use 8 orientation parameters to describe the orientation distribution of all the fiber. The next crucial step is to deduce the intensity distribution on the intersection plane between QS*(110)* and Ewald spheres, which will give us theoretical $I(\chi)$ profiles of *(110)* diffraction:

$$I(\chi) = \int_{-\frac{\pi}{2}}^{\frac{\pi}{2}} w(\gamma;\gamma_0,\Delta\gamma_0)\frac{1}{\sqrt{2\pi}a_x}\exp(-\frac{1}{2}(\frac{q_x(\chi;\alpha_0,\beta_0)\cos\gamma - q_z(\chi;\alpha_0,\beta_0)\sin\gamma}{a_x})^2)\mathrm{d}\gamma$$

For each fiber group, α_0 and β_0 are the two parameters indicating the out-of-plane tilt of the nanofibers in 3D, while γ_0 and Δγ indicating the main in-plane orientation and the spreading within the plane respectively (as shown in **Fig. 2)**. In this paper, we assume there are only two main fiber groups. Therefore, the orientation labels are composed of nine parameters, including three azimuthal parameters (α, β, γ), one angle spreading parameter ($\Delta\gamma$), as well as one quantity parameter λ indicating the quantity of that group for each nanofiber group. Because of indistinguishability between two groups of nanofibers in 3D space, thus herein, we define that γ label of the first group fibers is greater than that of the second to ensure unique mapping from a diffraction pattern to its corresponding orientation labels for ML algorithms. The orientation parameters of two groups of nanofibers are sampled from the corresponding intervals, summarized in Table S1.

In summary, for each group of nanofibers, there are four angular related parameters as well as a quantity parameter indicating its quantity. But because the quantity measurement for multiple groups of nanofibers is relative, we consider the quantity of one group of nanofibers as the standard, and the quantity of others groups of nanofibers is calculated relative to it. So, for two groups of nanofibers, there are a total of 5x2-1=9 parameters. Thereby, total targeted orientation labels are nine, including α, β, γ, $\Delta\gamma$ for each nanofiber group as well as the scale ratio (i.e., quantity ratio) for them. Figure S6 shows the distribution for them. The relationship between γ_1 *and* γ_2 is depicted in Figure S7. From sampling scheme here, $\boldsymbol{\lambda_1/\lambda_2}$ is distributed from 1/15 to 15.

Table S1 Sampling scheme and units of orientation parameters.

The labels, α_1, β_1, γ_1, $\Delta\gamma_1$, λ_1 are for group 1 and α_2, β_2, γ_2, $\Delta\gamma_2$, λ_2 for group 2. The notation "U" denotes the uniform distribution in the interval. The schematic definition for each group fiber is shown in Figure 2.

	**Sampling of parameters**	**Unit**
α_1	U(-5, 5)	degree
β_1	U(-90, 90)	degree
γ_1	U(-90, 90)	degree
$\Delta\gamma_1$	U(8, 89)	degree
λ_1	U(-450 / 15, 450)(p=1/2) & U(450, 450 * 15)(p=1/2)	dimensionless
α_2	U(-5, 5)	degree
β_2	U(-90, 90)	degree
γ_2	U(-90, 90)	degree
$\Delta\gamma_2$	U(8, 89)	degree
λ_2	450	dimensionless

The notation “U” denotes the uniform distribution in the interval.

Table S2 Hyperparameters used for various machine learning algorithms.

**Machine Learning Algorithms**	**Hyperparameters used**
K-nearest neighbors	n_neighbors: 6
Random forest	m_estimator: 300<br>max_depth: 35<br>max_features: 8<br>min_samples_leaf: 1<br>min_samples_split: 2
Support vector machine	kernel: rbf<br>RBF kernel coefficient: auto (that will be 1 / n_features)<br>epsilon-tube parameter: 0.001<br>Regularization parameter: 1.0
Fully connected neural networks	optimizer: Adam<br>learning rate: 0.0008<br>weight decay: 0.0<br>patience: 30
DenseNet	optimizer: Adam<br>learning rate: 1e-4<br>weight decay: 1e-7<br>patience: 30
PreActResNet	optimizer: Adam<br>learning rate: 1e-4<br>weight decay: 1e-7<br>patience: 30

Table S3 The reconstruction RMSE of FCNN trained under different types of noises settings on experimental dataset (1). random Poisson noises (using a random variable controlling its magnitude); (2) random Gaussian white noises of different magnitude; (3). considering both Poisson and Gaussian white noises.

**Poisson**		**Gaussian**		**Poisson and Gaussian**	
mean	SD	mean	SD	mean	SD
0.0902	0.0025	0.0982	0.0055	0.0987	0.0047

Table S4 The R^2 score of FCNN in the cases of indistinguishability and artificial distinguishability through γ magnitude for two groups of fibers.

indistinguishable		artificially distinguishable	
mean	SD	mean	SD
0.2319	0.0071	0.8141	0.0050
-0.4327	0.0313	0.9797	0.0048
-0.0773	0.0212	0.9643	0.0030
0.2402	0.0098	0.9296	0.0014
0.2319	0.0096	0.8083	0.0011
-0.4319	0.0351	0.9733	0.0045
-0.1056	0.0155	0.9654	0.0034
0.2343	0.0074	0.9271	0.0013
-0.1308	0.0315	0.9263	0.0088

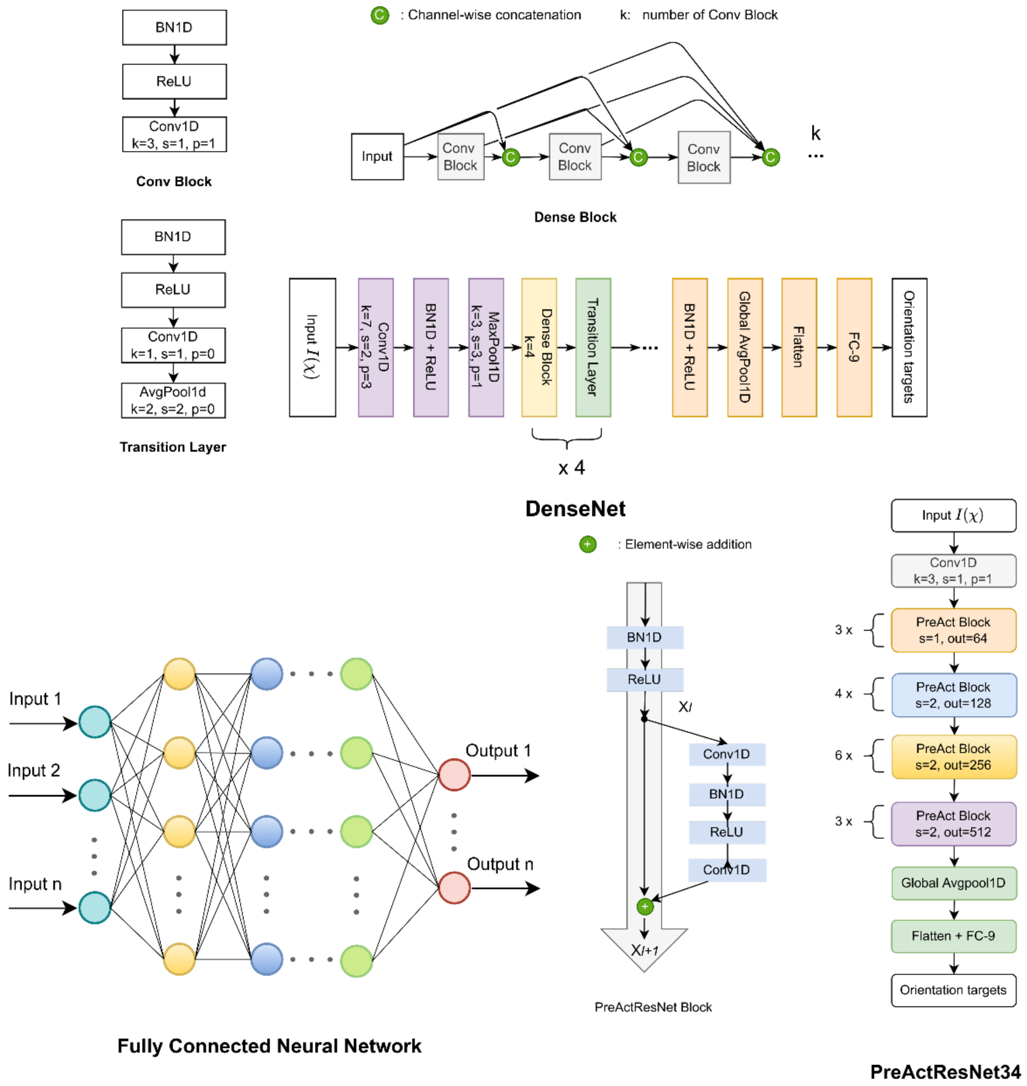

Figure S1 The architecture of DL algorithms, i.e., Fully connected neural network (FCNN), DenseNet and PreActResNet34.

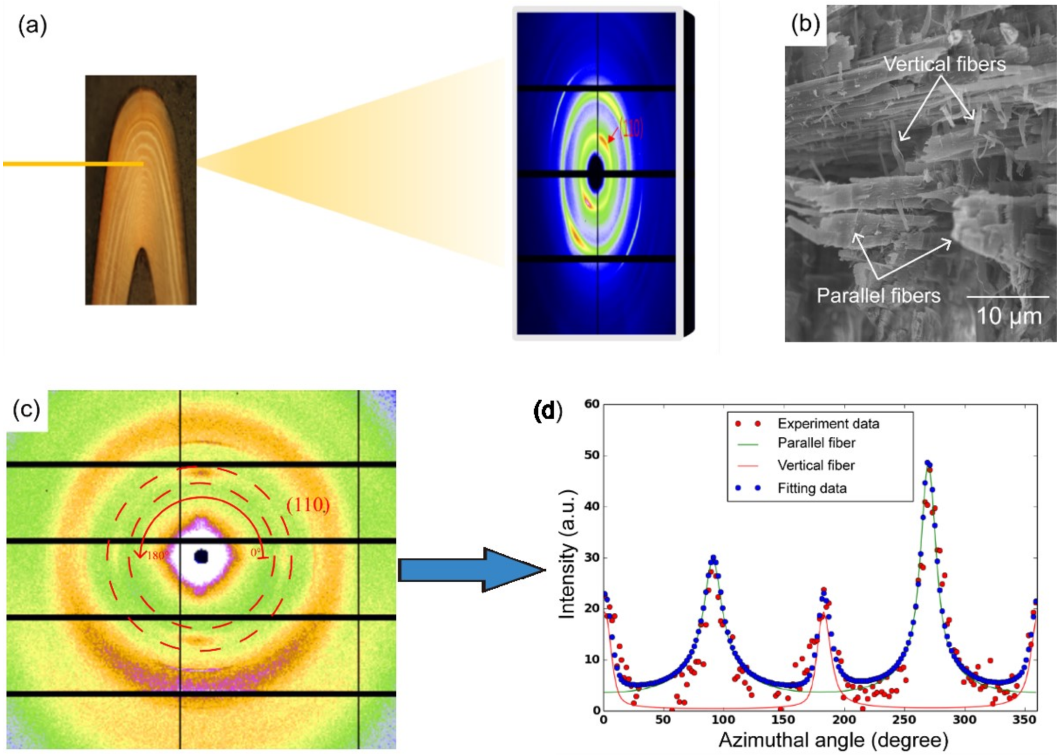

Figure S2 Experiment setup and data processing. (a) The light microscopy image of sample and the schematic of experiment setup for WAXD measurement. (b) The SEM image shows the vertical (out-of-plane) and the parallel (in-plane) fibers within the cuticle. (c) Experimental WAXD image with the *(110)* diffraction pattern located within the red dash rings. (d) Experimental and fitted I (χ) curves of the *(110)* diffraction.

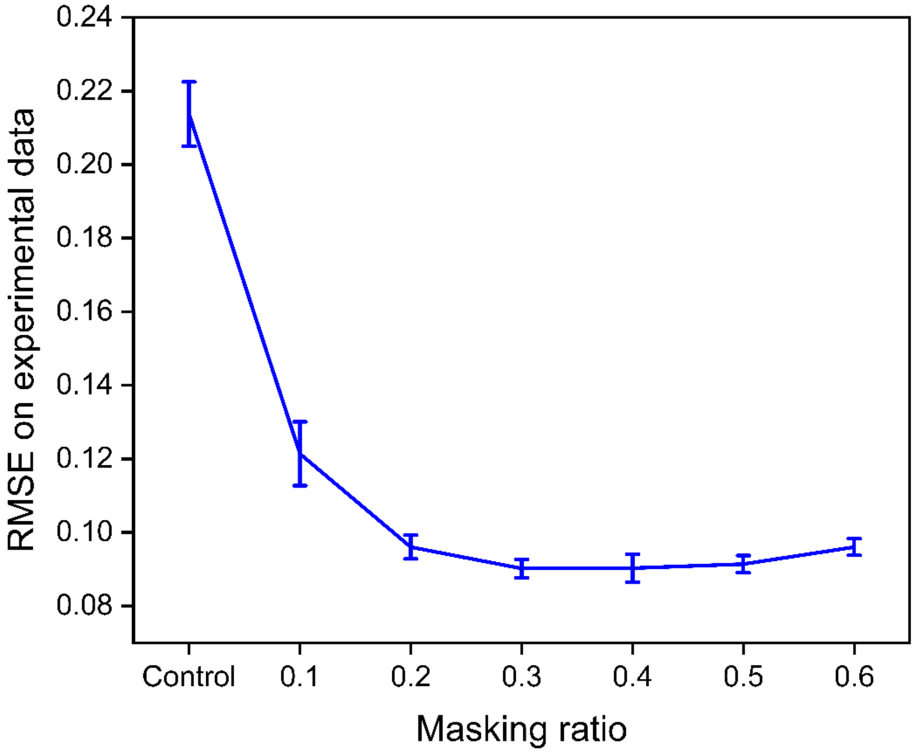

Figure S3 The reconstruction RMSE on experimental data vs. different masking ratio.

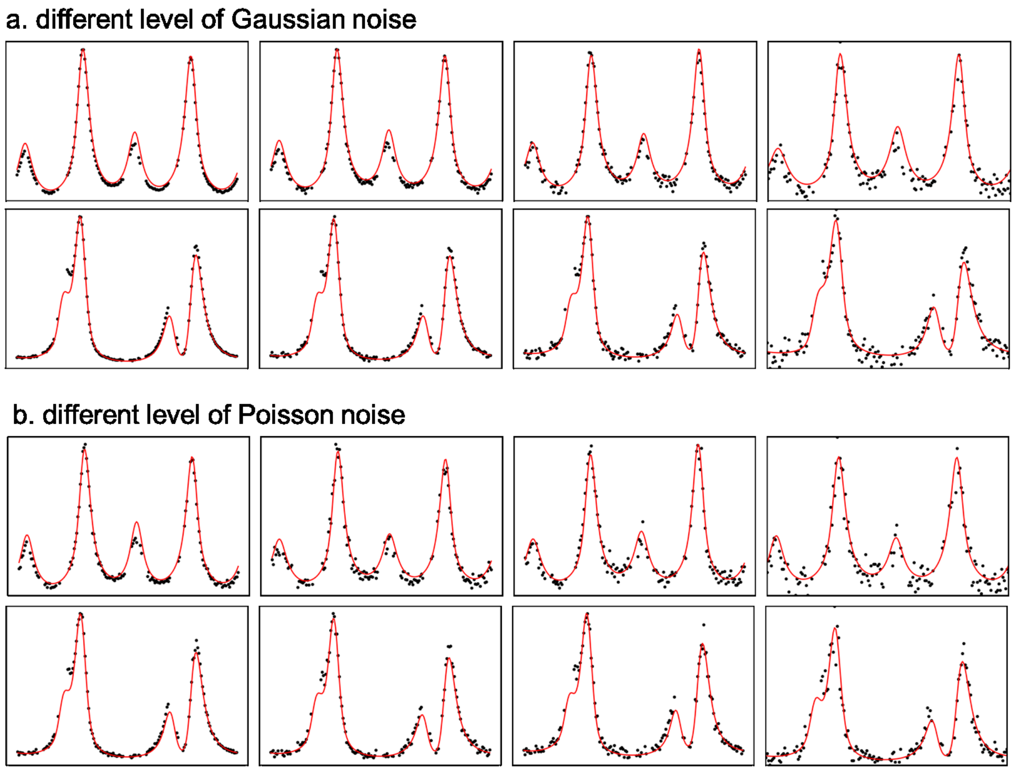

Figure S4 The reconstruction results for the I(χ) curves of different levels of SNR with (a) Gaussian and (b) Poisson noises.

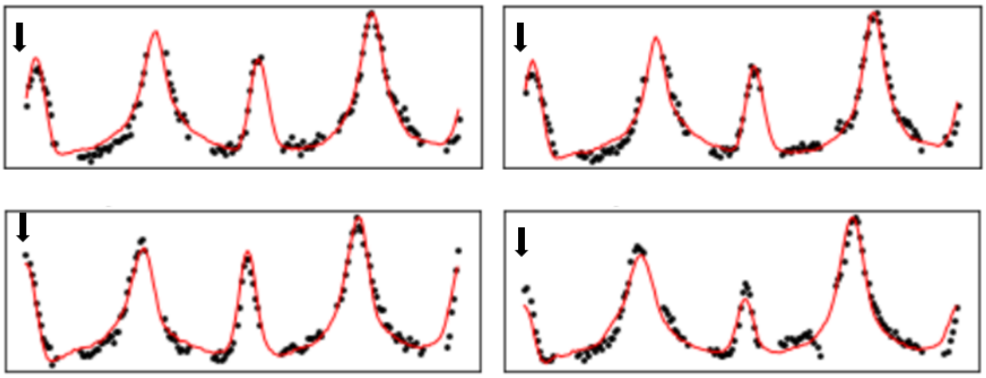

Figure S5 The reconstruction results of FCNN in the cases where the boundary emerges at different locations relative to diffraction peaks.

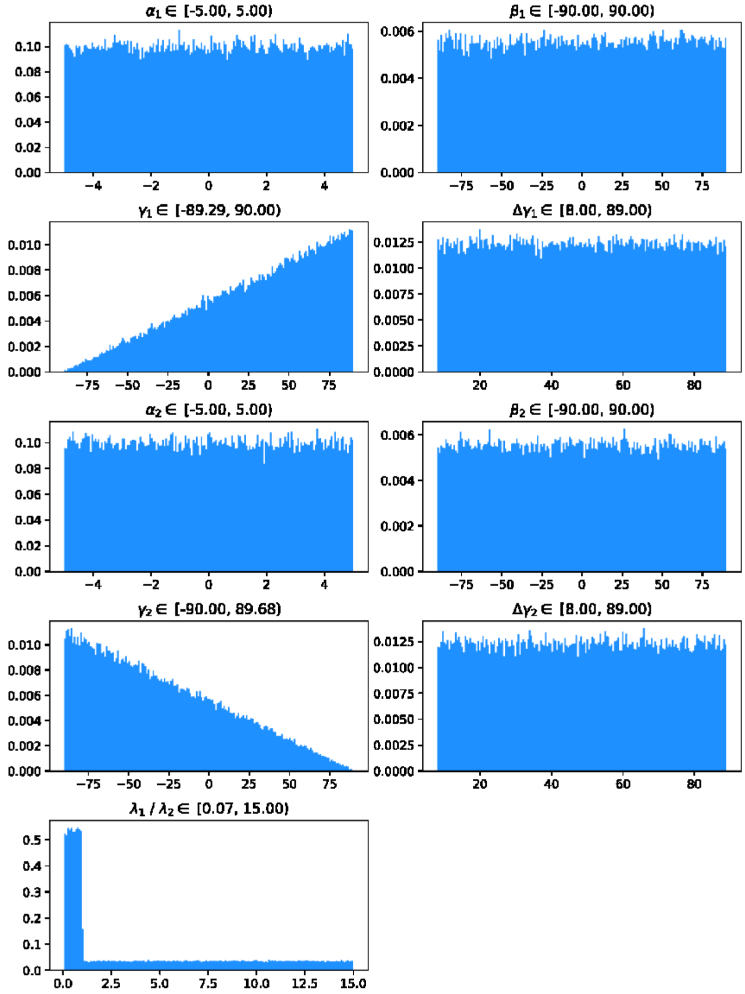

Figure S6 Orientation parameter distribution for simulated dataset.

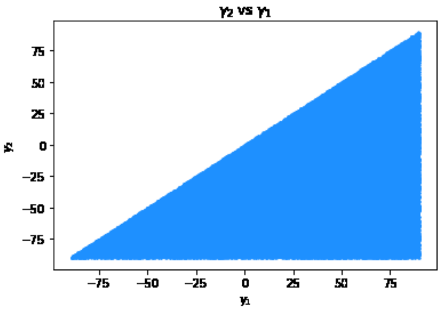

Figure S7 Relationship between γ_1 and γ_2 parameters. The γ_1 is greater than γ_2 so that two group of fiber can be distinguishable.

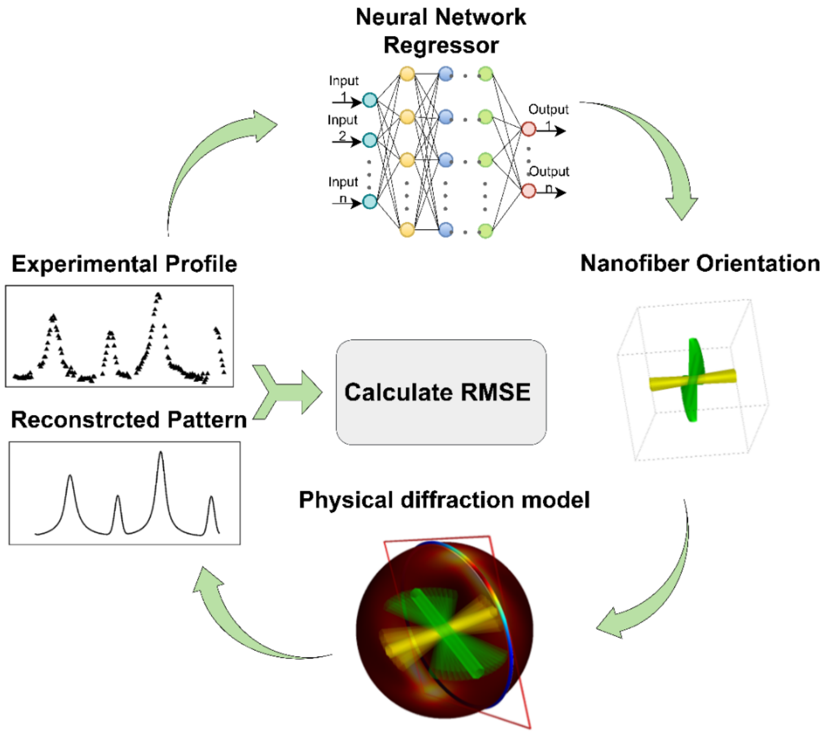

Figure S8 Root mean squared error (RMSE) calculation process between the normalized 1D experimental profile and the normalized reconstructed profile according to orientation prediction of ML algorithms through our developed in-house nanofiber diffraction model.

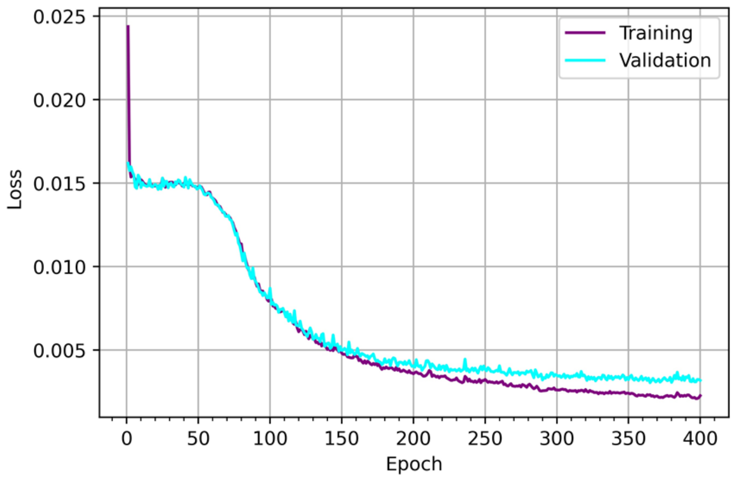

Figure S9 MSE loss curve of training and validation for training the FCNN.

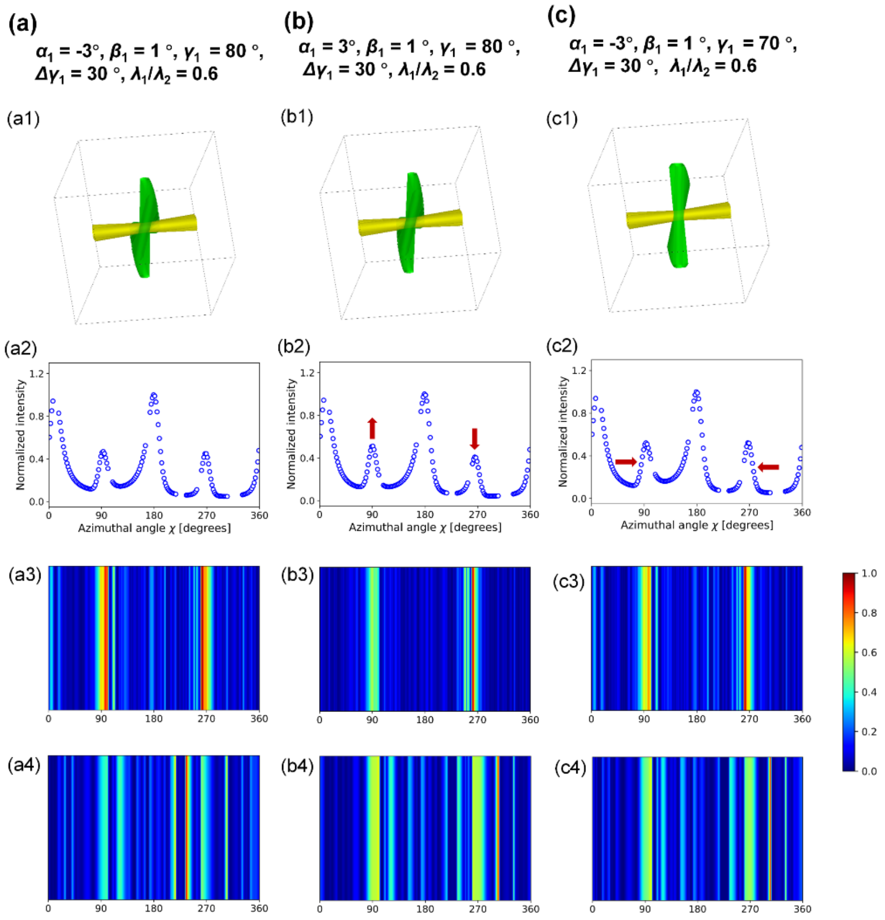

Figure S10 The saliency maps for α and γ, highlighting the features that FCNN model depends on. (a-d) the sketches of two fiber groups within the volume, with one orientation parameter of the specific fiber group (green) changes at each condition (a1-d1). (a2-d2) normalized simulation I(χ) profile corresponding to the parameter changes in a1-d1. (a3-d3) the saliency map with data corruption and (a4-d4) without data corruption.
